# Two major ruminant acute phase proteins, haptoglobin and serum amyloid A, as serum biomarkers during active sheep scab infestation

**DOI:** 10.1186/1297-9716-44-103

**Published:** 2013-10-31

**Authors:** Beth Wells, Giles T Innocent, Peter D Eckersall, Eilidh McCulloch, Alasdair J Nisbet, Stewart TG Burgess

**Affiliations:** 1 Moredun Research Institute, Pentlands Science Park, Midlothian EH26 0PZ, UK; 2 Biomathematics & Statistics Scotland (BioSS), King’s Buildings, Mayfield Road, Edinburgh EH9 3JZ, UK; 3 University of Glasgow, Sir Graeme Davies Building, 120 University Place, Glasgow G12 8TA, UK; 4 Reactivlab Ltd, Garscube Estate, Bearsden Rd, Glasgow G61 1QH, UK

## Abstract

Two ruminant acute phase proteins (APPs), haptoglobin (Hp) and serum amyloid A (SAA), were evaluated as serum biomarkers (BMs) for sheep scab–a highly contagious ectoparasitic disease caused by the mite *Psoroptes ovis,* which is a major welfare and production threat worldwide. The levels of both APPs increased in serum following experimental infestation of sheep with *P. ovis*, becoming statistically significantly elevated from pre-infestation levels at 4 weeks post-infestation. Following successful treatment of infested sheep with an endectocide, Hp and SAA serum levels declined rapidly, with half lives of less than 3 days. In contrast, serum IgG levels which specifically bound the *P. ovis*-derived diagnostic antigen Pso o 2 had a half-life of 56 days. Taking into account pre-infestation serum levels, rapidity of response to infestation and test sensitivity at the estimated optimum cut-off values, SAA was the more discriminatory marker. These studies illustrated the potential of SAA and Hp to indicate current sheep scab infestation status and to augment the existing Pso o 2 serological assay to give disease-specific indications of both infestation and successful treatment.

## Introduction

Sheep scab, caused by the infestation of sheep skin with the highly contagious mite *Psoroptes ovis*, affects the productivity and welfare of sheep as it produces intensely pruritic lesions and wool loss. These factors, along with the high cost of treatment, mean this disease has significant economic implications for the sheep industries of affected countries [1]. In the UK, the incidence of the disease has increased to a level that it is now endemic [2] with the national annual prevalence estimated at > 7000 outbreaks [3]. This has resulted in the disease being made notifiable in a number of countries (e.g. in Scotland through the Sheep Scab (Scotland) Order 2010 [4]), which has renewed the focus on accurate diagnosis for effective control. Accurate diagnosis can be challenging - clinical signs in *P. ovis* infested sheep may be minimal during early infestation (up to several months in the field) and easily missed but these animals represent a source of infestation [5]. In such a situation, mites can be difficult to locate and the sensitivity of the microscopic detection of mites in skin scrapings from these animals can be as low as 18% [6]. In addition to *P. ovis*, other ectoparasites such as ticks and lice can induce skin reactions which may appear similar to those seen in early infestation with *P. ovis*[7,8] and dual infestations with more than one ectoparasite are common. The difficulties highlighted above in the control and diagnosis of sheep scab illustrates the requirement for a sensitive and specific test which would indicate early infestation and also current disease status.

Several immunoassays have been developed to detect serum antibodies with specific reactivity to *P. ovis* antigens from infested sheep [9-11]. Recently we have successfully developed a diagnostic test which detects the host’s antibody response to the *P. ovis* antigen Pso o 2. This test is highly specific and sensitive and can detect serum antibodies to Pso o 2 as early as two weeks post-infestation [12]. However, due to antibody persistence of several months in the serum, the test cannot distinguish between currently-infested and recently-(successfully) treated sheep [13]. This is an important factor in any control or eradication regime where demonstration of freedom from infestation following treatment forms part of legislation on movement restrictions etc. Therefore, an improved diagnostic test for sheep scab should combine the pathogen specificity and early indication of infestation (as provided by the Pso o 2 ELISA [12]) with a biomarker (BM) element to differentiate between successfully-treated animals and those with active disease.

Ideally, BMs should circulate at low levels in the serum of healthy individuals, increase in titre rapidly following the onset of disease and return to pre-infestation levels soon after successful treatment or disease resolution [14]. As this describes the behaviour of inflammatory proteins and, as sheep scab produces an acute inflammatory response within hours of infestation [15], we investigated two ruminant acute phase proteins (APPs)–haptoglobin (Hp) and serum amyloid A (SAA)–as BMs for sheep scab. Ruminants are unique in that Hp is a major APP [16] and in sheep, as for cattle, APP production continues in chronic as well as acute infections, with roles in tissue repair. In cattle, Hp is synthesised only in response to inflammation [17] and Hp levels have been used extensively to monitor inflammatory disease [18] but to date there has been very little work on the acute phase response in sheep [16].

This study investigates qualitative and quantitative serum levels of Hp and SAA in sheep during experimental infestations with *P. ovis* and the effects of successful treatment on these APPs; reports Hp and SAA levels during other common conditions and diseases of sheep and in sheep diagnosed as clinically positive during a natural outbreak of sheep scab.

## Materials and methods

### Samples used in APP evaluation

#### Time course trial

To supply sera for initial immunoblot analyses, blood was sampled from each of 6 Scotch Mule mixed sex 1-2 year old sheep, by venous extraction, prior to infestation with *P. ovis.* Following infestation on the withers with approximately 50 mites, blood was sampled weekly from each animal for 6 weeks and sera were prepared by centrifuging whole blood at 900 *g* for 10 min. Sera were then stored at −20 °C until use. This part of the study was termed the “time-course trial” (TCT).

### Primary infestation and treatment trial

For the quantitative analysis of Hp and SAA, sera were obtained from 12 *P. ovis*-naïve, Scotch Mule, mixed sex, 1-2 year old sheep infested with *P. ovis* mites as described above. Blood samples were acquired before exposure and then weekly over a 6 week period post-infestation. At 6 weeks post-infestation, all twelve sheep were treated with an injectable endectocide (Dectomax, Pfizer Animal Health) by intramuscular injection at a dose of 1 mL per 33 kg bodyweight and blood was then sampled twice weekly for 2.5 weeks; then at 4, 9 and 14 weeks post-treatment. This part of the study was termed the “primary infestation and treatment” (PIT) trial.

### Secondary infestation and treatment trial

Fourteen weeks post-treatment the sheep were re-infested with *P. ovis* and blood samples were taken at 24 hours post-infestation then weekly for a further 6 weeks. On week 6, sheep were treated with endectocide again, as described above, and blood was then sampled twice weekly for a further 2.5 weeks. This part of the study was termed the “secondary infestation and treatment” (SIT) trial. Sera were prepared from each of the blood samples by centrifuging whole blood at 900 *g* then sera were stored at −20 °C until use. Lesion areas were measured at the time of each blood sampling post-infestation until 2 weeks post-treatment by measuring the length and width of the main lesion on each sheep and recording the result as an average mean lesion area (cm^2^) ± SEM.

### Field acquired infestations

In addition to these sera from experimental infestations, sera were also acquired from sheep with field-acquired sheep scab infestations (*n* = 12). Sera were available for these animals pre-infestation, at point of clinical diagnosis and two months post-treatment [13].

### Other common infections

To test the specificity of the Hp and SAA response, the samples listed in Table 1 were used to analyse Hp and SAA levels during other common infections and conditions of sheep.

**Table 1:** Sera samples used to test APP responses during common infections and conditions of sheep

**Infection / condition**	**Number of samples**
Early gestation^a^	10
Late gestation^b^	10
Gastro-intestinal nematodes^c^	6
Liver Fluke (*Fasciola hepatica*)^d^	6
Sucking lice (*Linognathus spp*.)^e^	4
Chewing lice (*Bovicola ovis*)^e^	4
Orf^f^	6
Johnes disease^g^	6

^a^Early gestation was within 1 week of conception and ^b^late gestation was 1 week prior to expected lambing date (samples from Moredun Research Institute (MRI)). ^c^Samples from sheep with field-acquired gastro-intestinal nematode (GIN) infections were from Firth Mains Farm, UK (MRI); species included *Teladorsagia circumcincta* and *Nematodirus battus.*^d^Samples were taken from UK sheep with field-acquired infections of liver fluke (samples provide by Dr H McDougall, MRI); ^e^samples from lice infected but *P. ovis*-uninfested sheep (provided by Prof. N. Sargison, R(D)SVS, UK; ^f^samples from sheep infected experimentally with orf virus or ^g^*Mycobacterium avium* subspecies *paratuberculosis* (MRI).

### Qualitative analyses of the APPs: Haptoglobin (Hp) and Serum Amyloid A (SAA)

Sera from each of 6 sheep in the TCT study were pooled according to time point post-infestation and were then diluted 1:10 with dH₂O. Polyacrylamide gel electrophoresis (SDS-PAGE), under denaturing conditions, was performed using NuPage BisTris 4-12% polyacrylamide gels (Invitrogen) with MES buffer (Invitrogen) according to the manufacturer’s instructions.

Gels were stained in SimplyBlue Safestain (Invitrogen) following the manufacturer’s protocol. Bands which increased in intensity through the time course of infestation were excised from the gel and identified using matrix assisted laser desorption ionization (MALDI) at the Moredun Proteomics Facility: Excised bands were destained and subjected to reductive alkylation using DTT and iodoacetamide. Gel pieces were digested overnight at 37 °C in trypsin and digests analysed on an Ultraflex II MALDI-ToF-ToF mass spectrometer (Bruker Daltonics). The masses obtained were used for database searching with the MASCOT search engine using Swiss-Prot and local databases with a 50 ppm mass tolerance window. Significant matches from the Peptide Mass Fingerprint data were confirmed by MS/MS analysis using the search criteria above and an MS/MS tolerance window of 0.5 Da.

Immunoblotting was performed using sera from individual sheep with different disease progression profiles in the TCT trial. Sheep 1 had a naturally-resolving lesion, which was at maximum size at week 4 post-infestation, whilst the lesion from Sheep 5 and 6 showed a gradual expansion over the time course of infestation. Sera from these sheep from each time point in the TCT trial were separated by SDS-PAGE as described above and then transferred to a nitrocellulose membrane by electroblotting using the iBlot Western Blotting system (Invitrogen), following manufacturer’s instructions. The blot was blocked using 3% gelatin from cold water fish skin (Aldrich) for 30 min prior to washing with washing buffer/antibody diluent (10% PBS; 90% dH₂O, 29.22 g NaCl and 5 mL Tween 80 (Sigma-Aldrich). The blot was incubated with the following antibodies for 1 h with washing in between incubations: The primary antibody used for detecting Hp in the electrophoresed serum samples was a rabbit polyclonal anti-human Hp (Abcam, ab85846) used at a concentration of 1 μg/mL. The secondary antibody conjugate used was a swine anti-rabbit IgG HRP conjugate (Dako, P0399) used at a concentration of 0.5 μg/mL. Visualisation was by ECL Plus (GE Healthcare) using the ImageQuant system (GE Healthcare). The primary antibody for the detection of SAA was a rabbit polyclonal anti-human recombinant SAA (Abcam, ab59736) used at a concentration of 2 μg/mL. The conjugate was a swine anti-rabbit HRP IgG (Dako P0399) used at a concentration of 0.5 μg/mL. Band densities were estimated using the Quantity One software 4.6.2 (Bio-Rad).

### Quantitative analyses of the AAPs

Hp concentrations were determined by ReactivLab and Glasgow University using a colorimetric assay (ReactivLab Ltd, Wetherby, UK) based on the method described by Eckersall et al. [19] and modified as described in International Patent Application WO 2012/085497 A1. Validation of this cross species assay for Hp was described in Crawford et al. [20]. For determination of the concentration of Hp in ovine serum the assay was further validated. The intra-assay coefficient of variance (CV) was 5.5% calculated as the mean of CVs of samples (*n* = 47) run in duplicate in one assay, the inter-assay CVs using quality control samples of ovine serum were 6.5% at a mean Hp of 0.34 g/L (*n* = 7), 5% at a mean Hp of 0.71 g/L (*n* = 7) and 11.5% at a mean Hp of 1.01 g/L (*n* = 5). The limit of detection was 0.02 g/L determined as the concentration of Hp at 3 standard deviations from the mean of a blank sample. Hp concentrations were quantified in sheep sera from the PIT and SIT trials; from the field-acquired *P. ovis* infestations and from sheep with other common conditions (Table 1). The Hp assay was performed on an ABX Pentra 400 analyser (Horiba Medical) using a calibration curve with a top Hp standard of 1.48 mg/mL such that samples with Hp values greater than this were automatically diluted. All samples were tested in duplicate.

For SAA, a commercially-available ELISA kit (TP-802, Tridelta Development Ltd) was used to quantify levels of this APP in sheep sera. The manufacturer’s protocol was followed using the same sera samples as described above for Hp. Samples were tested from individuals in duplicate alongside a SAA standard curve and all serum was diluted 1:500 in sample diluent buffer (Tridelta Development Ltd). The upper level of the range of the bovine standards used was 300 ng/mL therefore all sera samples showing SAA values greater than this were further diluted as required and re-analysed.

### Analysis of serum IgG levels to the mite antigen Pso o 2

An ELISA which measures the levels of Pso o 2-specific IgG in sheep serum [12] was used to allow post-infestation comparisons of Pso o 2-specific IgG synthetic and decay profiles (post-treatment) with those of Hp and SAA. Serum samples from the PIT and SIT experimental trials were used to assess persistence in antigen-specific IgG levels after treatment [11,13]. Serum samples were analysed up to, and including, 14 weeks post-treatment. The ELISA was performed as previously described [12] with the following modifications: all sera samples were assayed for individual sheep in duplicate. Positive, inter-plate controls were used from sheep (*n* = 2) which had been re-infested with *P. ovis* for 6 weeks following a primary infestation (6 weeks) and effective treatment. Negative controls consisted of the pre-infestation sera from the PIT trial sheep.

### Statistical analysis

Initial statistical analyses of Hp and SAA levels in the PIT and SIT trials were performed using repeated measures one-way analysis of variance (ANOVA) with a Tukey’s post-hoc test and performed in Graph Pad Prism (Version 5.05, GraphPad Software Inc). Further statistical analyses were performed by Biomathematics and Statistics Scotland (BioSS) using the R Statistical Package [21]: Best fit models were identified to describe Hp, SAA and Pso o 2-specific IgG trends (as “normal” or “elevated” compared to pre-infestation levels) over the time course of infestation and post-treatment; half life values were calculated using a generalised linear mixed model to describe the exponential decay of the BMs and Pso o 2-specific IgG and incorporated a random effect of animal on the decay constant, and the initial value. In addition, optimised cut-off values were calculated, where the objective was to obtain as high sensitivity and specificity as possible to keep false results to a minimum. This was achieved using a generalised linear mixed model for binomial data, with a logit link which required the use of the library “lme4” [22].

## Results

### Qualitative analyses of the APPs: Hp and SAA from the TCT trial

**Figure 1:**
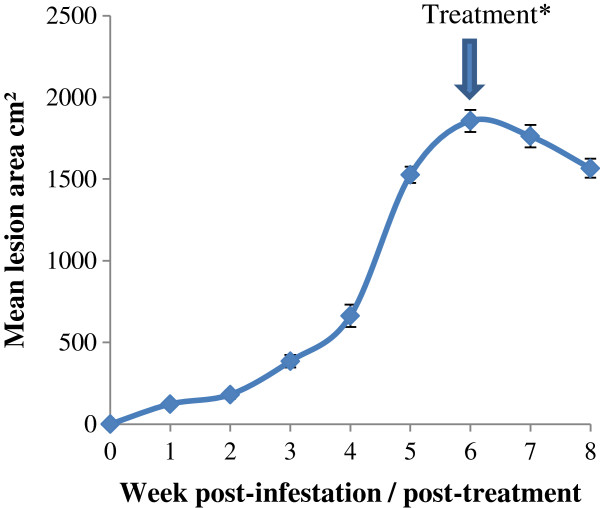
**Skin lesion areas for sheep over a 6 week time course of infestation with *Psoroptes ovis *and 2 weeks post-treatment (PIT trial data).** *Values shown are means (± SEM, *n* = 12). All animals treated with Dectomax (Pfizer Animal Health) at 6 weeks post-infestation.

Mean lesional areas are shown in Figure 1 and illustrate gradual lesion growth until week 4 post-infestation, followed by rapid expansion between week 4 to week 6 post-infestation. Post-treatment, the lesions resolved slowly and measurement was stopped 2 weeks after treatment due to the nature of lesion healing as they healed and lifted off rather than decreased in size.

**Figure 2:**
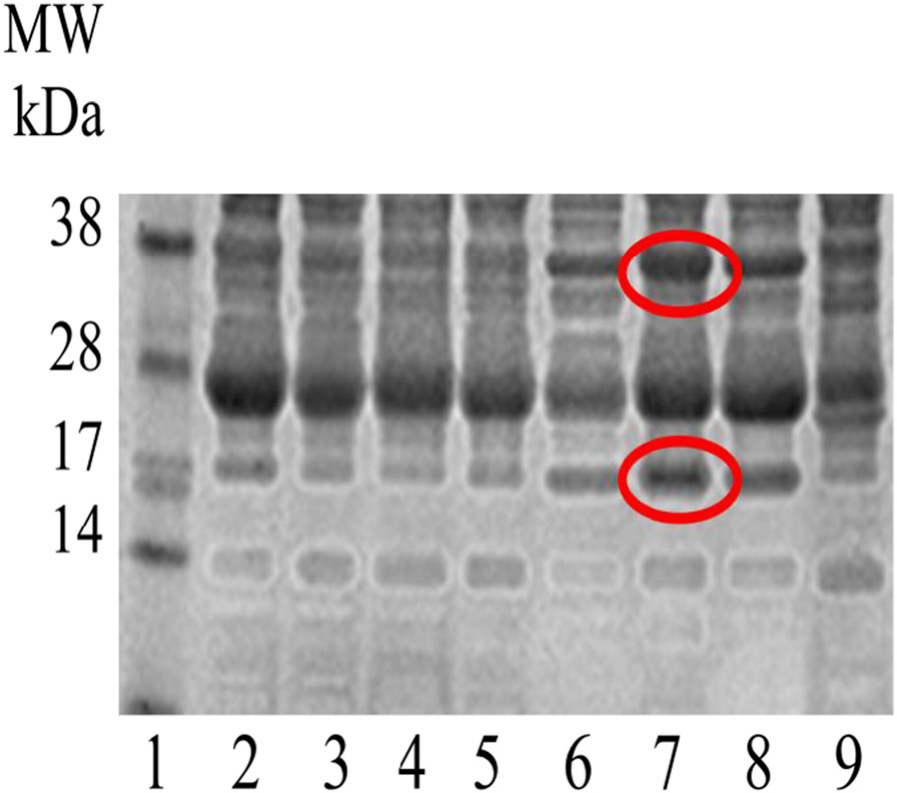
**Profile of serum proteins from sheep infested with *P. ovis *over a 6 week time course (TCT trial).** Lane 1 = SeeBlue Marker (Invitrogen); Lane 2 = pre-infestation sera; Lane 3 = week 1 post-infestation sera; Lane 4 = week 2 post-infestation sera; Lane 5 = week 3 post-infestation sera; Lane 6 = week 4 post-infestation sera; Lane 7 = week 5 post-infestation sera; Lane 8 = week 6 post-infestation sera; Lane 9 = hyper immune sera. Gel stained with SimplyBlue Safestain (Invitrogen). Bands excised for MALDI analysis at 17 and 38 kDa are circled in red.

The densities of two bands, with approximate molecular masses of 17 and 38 kDa, increased through the time course of infestation with *P. ovis,* with maximum abundance at weeks 5 and 6 post-infestation (Figure 2).

MALDI analysis identified both bands as fragments of Hp. As the ovine Hp sequence has not yet been deposited in public databases, the polypeptide with highest homology to the 17 kDa band was *Capra ibex* Hp with sequence coverage of 35.7% and intensity coverage of 79.7% (mascot score 127). For the 38 kDa sample, the polypeptide with highest homology was *Cervus elaphus* Hp with an intensity coverage of 11.3% and sequence coverage of 14.5% (mascot score 202).

**Figure 3:**
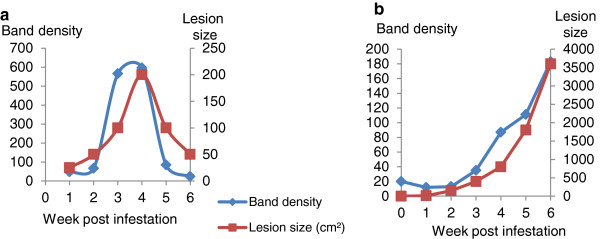
**Relationship between lesion area and serum Hp during a 6 week infestation of sheep with *Psoroptes ovis.*** Panel **a**: Serum from a sheep infested with *P. ovis* in trial TCT where the lesion resolved naturally after 4 weeks (Sheep 1); Panel **b**: Serum from an infested sheep in the same trial where the lesion had continued to expand during the 6 week time course (Sheep 5). Immunoblots were probed with a rabbit polyclonal anti-human Hp antibody (Abcam) and the secondary antibody conjugate used was a swine anti-rabbit IgG HRP (Dako). The image was visualised by ECL Plus and ImageQuant. Band volumes (at 45 kDa) were quantified by ImageQuant TL software.

**Figure 4:**
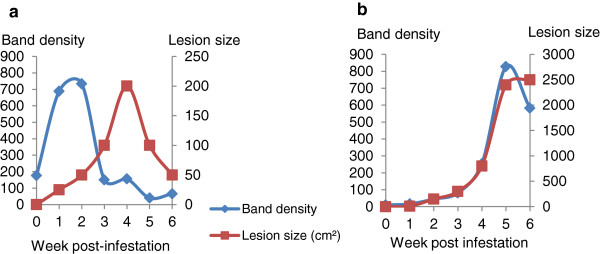
**Relationship between lesion area and serum SAA during a 6 week infestation of sheep with *Psoroptes ovis.*** Panel **a**: Serum from a sheep infested with *P. ovis* in trial TCT where the lesion resolved naturally after 4 weeks (Sheep 1); Panel **b**: Serum from an infested sheep in the same trial where the lesion had continued to expand during the 6 week time course (Sheep 6). The blots were probed with an anti-SAA antibody raised in rabbits against human rSAA (Abcam) and the secondary antibody conjugate was swine anti-rabbit HRP IgG (Dako). The blot was visualised using ECL Plus (GE Healthcare) and ImageQuant. Band volumes (at 14 kDa) were quantified by ImageQuant TL software.

Using immunoblot and densitometry analysis, along with lesion area data from trial TCT, it was demonstrated that, as the lesion size increased, the band density of Hp (at 45 kDa) also increased and as the lesion resolved the intensity of the bands decreased (Figure 3). For SAA, the densities of bands obtained in immunoblots, at the estimated MW of SAA (14 kDa), were also measured (Figure 4) and a clear relationship between SAA band density and lesion size was identified, with SAA band density peaking 2 weeks before the lesion size peak in the lesion resolving sheep (Figure 4a).

### Quantitative analyses of the APPs

**Figure 5:**
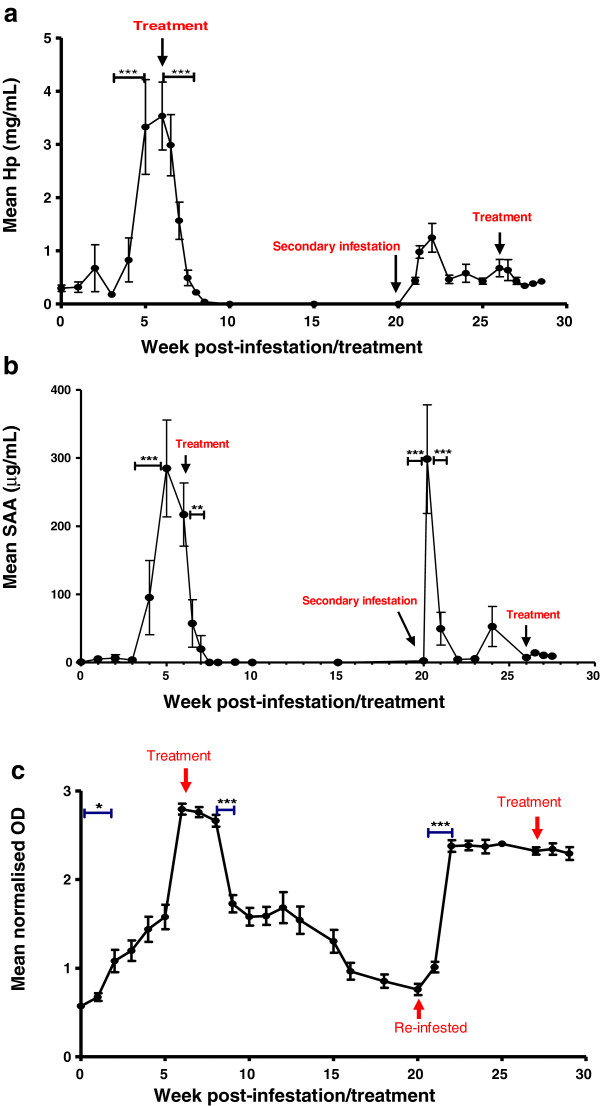
**Quantitative analyses of serum acute phase protein and Pso o 2-specific IgG levels during an infestation/treatment/re-infestation model of *Psoroptes ovis *in sheep.** Panel **a**: Haptoglobin: Sera samples from individual sheep (*n* = 12) in the PIT and SIT trial were analysed in duplicate using a colorimetric assay (ReactivLab). Hp concentrations are shown as mean (±SEM) through the time course of primary infestation (PIT) with *P. ovis* for 6 weeks, and post-treatment over 14 weeks, followed by secondary infestation (SIT) for a further 6 weeks and post-treatment for 2.5 weeks. Panel **b**: SAA: Sera samples from individual sheep (*n* = 12) in the PIT and SIT trial were analysed in duplicate using SAA ELISA (Tridelta Development Ltd). SAA concentrations are shown as mean (± SEM) through the time course of primary infestation (PIT) with *P. ovis* for 6 weeks, and post-treatment over 14 weeks, followed by secondary infestation (SIT) for a further 6 weeks and post-treatment for 2.5 weeks. Panel **c**: Pso o 2-specific IgG: Sera samples from individual sheep (*n* = 12) in the PIT and SIT trial were analysed in duplicate using an ELISA in which the coating antigen was a recombinant Pso o 2 (rPso o 2) at 75 μg/mL; primary antibody was IgG in the serum samples and the conjugate was a rabbit anti-sheep IgG HRP (Dako) at a dilution of 1:2000. IgG levels are represented as mean normalised OD₄₅₀ values (±SEM). *p ≤ 0.05; ** *p* ≤ 0.01 and ***p ≤ 0.001 as determined by repeated measures one-way ANOVA (Graph Pad Prism).

The mean Hp concentration in sheep serum pre-infestation with *P. ovis* was 0.30 ± 0.06 mg/mL. There was no statistically significant increase in Hp levels at the 5% level during the primary infestation until after 4 weeks post-infestation (Figure 5a). Between weeks 4 and 5 post-infestation, mean Hp levels increased from 0.83 ± 0.41 mg/mL to 3.33 ± 0.89 mg/mL (*p* ≤ 0.001). Following termination of the primary infestation with endectocide, mean Hp levels fell significantly (*p* ≤ 0.001) from 3.53 ± 0.64 mg/mL at the point of treatment to 1.57 ± 0.35 mg/mL one week post-treatment and had returned to pre-infestation levels between 10 and 14 days after treatment. During this PIT phase of the trial, the proportion of animals with “elevated” Hp increased between weeks 4 and 5 post-infestation and all, except one, animal had returned to “normal” by 10 days post-treatment (Table 2). The half life of Hp levels in the serum post-treatment was calculated as 2.3 days [95% confidence interval (CI) = 1.9-2.9] and a cut-off of 1.26 mg/mL was selected, giving an estimated sensitivity of 0.83 and a specificity of 1.0.

**Table 2:** **Distribution of sheep into “normal” and “elevated” groups for serum Hp and SAA concentration across the time course of primary infestation with *Psoroptes ovis *** **and treatment with an endectocide (PIT trial)**

**Biomarker**	**Week**	**0**	**1**	**2**	**3**	**4**	**5**	6**	**6.5**	**7**	**7.5**	**8**
**Hp**	Normal*	12	11	11	11	10	4	2	2	7	11	12
	Elevated	0	1	1	1	2	8	10	10	5	1	0
**SAA**	Normal	12	9	10	11	4	0	0	7	11	12	12
	Elevated	0	3	2	1	8	12	12	5	1	0	0

* The status of APP levels in sheep sera (*n* = 12 for each time point) was determined by comparison to pre-infestation levels using the R Statistical Package.

** All sheep were treated with an injectable endectocide at week 6 post-infestation. In these analyses the animals were described as being “normal” as in day 0 (pre-infestation) or “elevated”. As the results showed a normal distribution, all values above the 0.999 quantile of the distribution were considered to be elevated, giving a 99.9% specificity and undefined sensitivity.

The Hp profile during the secondary infestation (Figure 5a) showed that serum Hp levels increased more rapidly following this secondary challenge than during the primary infestation with *P. ovis.* Hp levels had doubled by 24 hours post-infestation in the secondary infestation compared with the much slower increase in levels during the primary infestation. The maximum Hp concentration measured during the secondary infestation was at 2 weeks post-infestation (week 22) with a value of 1.25 ± 0.27 mg/mL. By week 3 in the secondary infestation, Hp levels had decreased to pre-infestation levels and remained at low levels for the remainder of the trial.

SAA levels pre-infestation were 0.82 ± 0.53 μg/mL and remained at this level over the first 3 weeks of primary infestation (PIT, Figure 5b). By week 4 post-infestation, SAA levels had increased to 95.24 ± 54.4 μg/mL and thereafter peaked at week 5 (284.75 ± 71.144 μg/mL). The increase in serum SAA levels between weeks 3 and 5 post-infestation was highly statistically significant (*p* ≤ 0.001). Post-treatment, serum SAA levels fell within 3 days of treatment to 57.28 ± 34.75 μg/mL (*p* ≤ 0.05) and continued to decrease reaching pre-infestation levels within 10 days post-treatment. The proportion of animals showing elevated serum SAA increased between weeks 4 and 5 post-infestation and all except one animal had returned to normal by 10 days post-treatment (Table 2). The half life of SAA in the serum post-treatment was estimated at 0.84 days (95% CI = 0.73-0.99) and a cut-off value of 29.5 μg/mL was selected to identify “elevated” animals above that point or “normal” animals below it. This cut-off value provided an estimated assay sensitivity of 1.0 and a specificity of 1.0.

The secondary infestation was characterised by a rapid increase in SAA concentration of the same magnitude of the primary infestation peak, but within 24 h post-secondary infestation. Within 2 weeks of infestation, however, SAA levels had fallen to pre-infestation levels (2.26 ± 2.46 μg/mL) and they remained at this level until the end of the trial.

### Analysis of serum IgG levels to the mite antigen Pso o 2

By week 2 post-primary infestation (PIT trial) the mean normalised OD₄₅₀ value measured in ELISAs to detect Pso o 2-specific IgG in sheep serum had increased from 0.57 ± 0.02 pre-infestation to 1.197 ± 0.115 (*p* ≤ 0.05) and continued to increase until week 6 post-primary infestation (Figure 5c). The antigen-specific IgG levels increased, exponentially, over time during the primary infestation period up to the point of treatment by 0.034 OD_450_ units per day (95% CI = 0.032-0.037). By week 3 post-infestation, 9 out of 12 animals showed an “elevated” Pso o 2-specific IgG level (Table 3). After termination of the primary infestation with endectocide, the mean OD₄₅₀ decreased from a 2 weeks post-treatment value of 2.66 ± 0.07 to 1.73 ± 0.10 by 3 weeks post-treatment (*p* ≤ 0.001). After this initial decline, the OD_450_ values continued to decline slowly and did not return to pre-infestation levels until the start of the secondary infestation trial, which was 14 weeks post-treatment. The half life of the antibody levels to Pso o 2 following endectocide treatment was estimated as being 56 days with a decay rate of 0.012 OD_450_ units per day (95% CI = 0.011-0.014). More than half of the sheep in the PIT trial continued to show elevated Pso o 2-specific IgG levels at 14 weeks post-treatment. As the anti-Pso o 2 antibody levels increased at approximately three times the rate at which they decreased, it is evident that antibody levels were not an accurate measure of current disease status post-treatment, but were effective during the early diagnosis of sheep scab during this trial.

**Table 3:** **Distribution of sheep into “normal” and “elevated” groups for Pso o 2-specific serum IgG across the time course of a primary infestation with *Psoroptes ovis *** **and treatment with an endectocide (PIT trial)**

**Week**	**0**	**1**	**2**	**3**	**4**	**5**	6*	**7**	**8**	**9**	**10**	**11**	**12**	**13**	**14**	**15**	**16**	**17**	**18**	**19**	**20**
Normal	12	9	4	3	1	1	0	0	0	0	0	0	0	1	1	1	2	4	5	5	6
Elevated	0	3	8	9	11	11	12	12	12	12	12	12	12	11	11	11	10	8	7	7	6

* All animals were treated with injectable endectocide at week 6 post-infestation. In this analysis the animals were described as being “normal” as in day 0 (pre-infestation) or “elevated”. As the results showed a normal distribution, all values above the 0.999 quantile of the distribution were considered to be elevated, giving a 99.9% specificity and undefined sensitivity.

### Assessment of APP levels in sheep during a field outbreak of sheep scab

**Figure 6:**
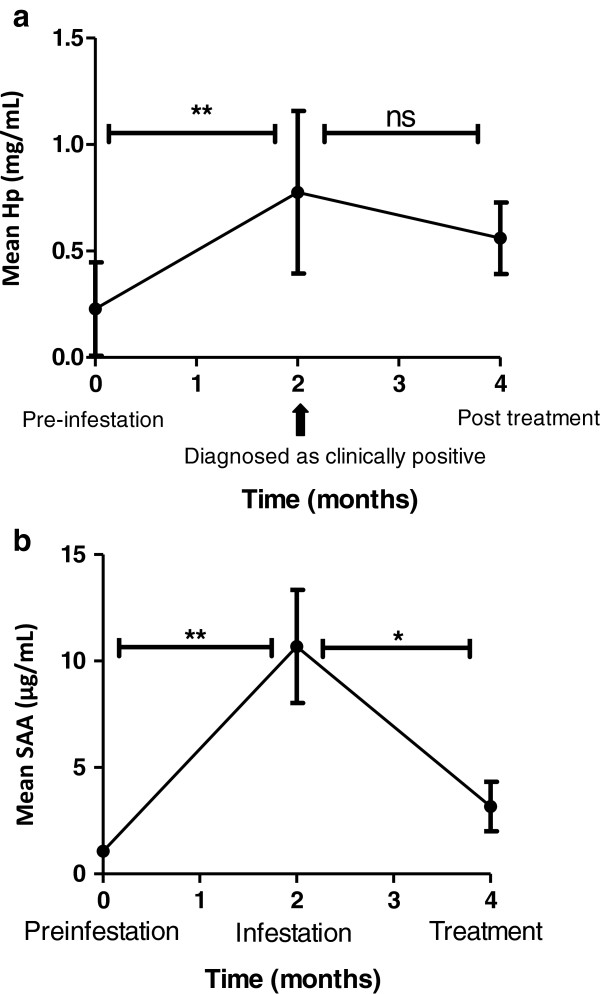
**Quantitative analyses of serum acute phase proteins in sheep during a field-acquired infestation with *Psoroptes ovis*.** Mean serum Hp (Panel **a**) and SAA (Panel **b**) concentrations (± SE) from sheep (*n* = 12) pre-infestation, at the point of clinical diagnosis of sheep scab and 2 months post-treatment with an endectocide in a field outbreak of sheep scab. Hp measurement by commercial assay (ReactivLab) and SAA by commercial ELISA (Tridelta Development Ltd); sera from individual sheep diluted 1:50 for SAA assay and assayed in duplicate in both assays. **p ≤ 0.01; *p ≤ 0.05 as determined by a one-way ANOVA (Graph Pad Prism).

The pre-infestation levels of Hp (0.23 ± 0.22 mg/mL) were comparable to those, pre-infestation, in the PIT experimental trial (0.29 ± 0.06 mg/mL) (Figure 6a). Following a field-acquired infestation with *P. ovis*, serum Hp levels were statistically significantly elevated at the point of clinical diagnosis (*p* ≤ 0.01) but the decrease in Hp levels 2 months post-treatment was not statistically significant.

The pre-infestation levels of SAA (1.07 ± 0.24 μg/mL) in these sheep (Figure 6b) were marginally higher than those in the PIT experimental trial (0.41 ± 0.26 μg/mL). At point of clinical diagnosis the recorded mean SAA (10.69 ± 2.66 μg/mL) was statistically significantly higher than the pre-infestation level (*p* ≤ 0.01) and, unlike Hp, the decrease in SAA post-treatment was also statistically significant (*p* ≤ 0.05, Figure 6b).

### Hp and SAA levels during other common sheep infections and conditions

The serum Hp and SAA levels measured during most other common sheep infections were similar to those measured in sheep prior to infestation with *P. ovis* (Table 4). For example Hp and SAA levels during experimental gastro-intestinal nematode infections were 0.04 ± 0.03 mg/mL and 0.30 ± 0.01 μg/mL respectively. The exceptions to this were during liver fluke infection and Johnes disease, where Hp levels were 2.85 ± 0.02 mg/mL and 2.25 ± 0.34 mg/mL respectively, which are of the same magnitude as those measured 4-5 weeks post-infestation with *P. ovis.* SAA levels during chewing and sucking lice infestations also showed the same magnitudes as 4-5 weeks post-infestation with *P. ovis* at 193.36 ± 135.81 μg/mL and 68.47 ± 22.33 μg/mL respectively.

**Table 4:** Serum Hp and SAA concentrations during common sheep diseases and conditions

**Infection/Condition**	**Mean Hp (mg/mL)**	**Mean SAA (μg/mL)**
Early gestation^a^ (*n* = 10)	0.33 ± 0.01	9.34 ± 2.14
Late gestation^b^ (*n* = 10)	0.52 ± 0.08	17.09 ± 7.77
Gastro-intestinal nematodes^c^ (*n* = 6)	0.04 ± 0.03	0.30 ± 0.01
Liver Fluke (*Fasciola hepatica*)^d^ (*n* = 6)	2.85 ± 0.02	0.22 ± 0.01
Sucking lice (*Linognathus spp*.)^e^ (*n* = 4)	0.52 ± 0.51	68.47 ± 22.33
Chewing lice (*Bovicola ovis*)^e^ (*n* = 4)	0.65 ± 0.31	193.36 ± 135.81
Orf (*n* = 6)^f^	0.21 ± 0.01	0.93 ± 0.53
Johnes disease^g^ (*n* = 6)	2.25 ± 0.34	21.43 ± 10.47

Hp (mg/mL ± SEM) measured by colorimetric assay (ReactivLab) and SAA (μg/mL ± SEM) assessed by SAA ELISA (Tridelta Development Ltd). All samples were assayed for individual animals in duplicate. ^a^Early gestation was within 1 week of conception and ^b^late gestation was 1 week prior to expected lambing date (samples from Moredun Research Institute (MRI)). ^c^Samples from sheep with field-acquired gastro-intestinal nematode (GIN) infections were from Firth Mains Farm, UK (MRI); species included *Teladorsagia circumcincta* and *Nematodirus battus.*^d^Samples were taken from UK sheep with field-acquired infections of liver fluke (samples provide by Dr H McDougall, MRI); ^e^samples from lice infected but *P. ovis*-uninfested sheep (provided by Prof. N. Sargison, R(D)SVS, UK; ^f^samples from sheep infected experimentally with orf virus or ^g^*Mycobacterium avium* subspecies *paratuberculosis* (MRI).

## Discussion

Here we identified, by proteomic and semi-quantitative immunoproteomic techniques, serum Hp and SAA as potential biomarkers for active sheep scab infestation. The qualitative results for SAA in the lesion resolving sheep (Figure 4a) where the band densities increased then decreased around 2 weeks before the lesion size changes, suggested that SAA levels in the serum of infested sheep may increase before obvious clinical signs were apparent, which would be an important factor in the pre-clinical diagnosis of sheep scab. However, when the levels of SAA and Hp in sera were quantified using assays, it was demonstrated that both APPs increased in serum concentration as *P. ovis* infestation progressed, but did not show statistically significant increases (*p* ≤ 0.001) until weeks 4–5 post-infestation. Previous studies have described increases in serum Hp and SAA levels during a range of inflammatory diseases in ruminants [16,23,24], but this is the first report of their elevation during sheep scab disease.

During a six week experimental infestation of sheep with *P. ovis*, Hp levels in serum increased by more than 10-fold to 3.5 mg/mL compared with pre-infestation levels, whereas SAA levels increased approximately 1000-fold in the same period to 211 μg/mL. These levels were similar to those of Hp and SAA measured in Alpine ibex (*Capra ibex*) infested with clinical sarcoptic mange (*Sarcoptes scabiei* infestation) where mean Hp levels during naturally-acquired infestation increased to 3.72 ± 0.65 mg/mL compared with healthy, uninfested controls (0.58 ± 0.09 mg/mL) and SAA levels were 130.7 ± 0.16 μg/mL and 8.7 ± 0.13 μg/mL in infested and uninfested animals respectively [25]. Generally, major APPs are present at undetectable or very low levels in the serum of healthy animals [26] but, recently, a study investigating the APP response to scrapie in sheep found large individual animal variation in Hp and SAA prior to clinical disease onset and it was suggested that this may be due to underlying subclinical conditions [27]. Previous work has indicated that Hp and SAA are non-specific in terms of inflammatory disease, injury or infection, but highly sensitive, effective markers of inflammation in ruminants [16,24] suggesting that any inflammatory event in the animal may cause a temporary rise in Hp or SAA. In contrast to Hp, serum SAA was measured at lower levels in sheep serum prior to *P. ovis* infestation; mean SAA concentration pre-infestation during the PIT trial was 0.82 ± 0.53 μg/mL, representing only 0.29% of the peak SAA levels measured at week 5 post-infestation. This suggests that SAA may potentially be a more suitable BM than Hp to indicate current disease status during *P. ovis* infestation in sheep although the biochemical assay for Hp has practical advantages being an automated rapid assay which could be incorporated into routine biochemistry profile analysis in contrast to the ELISA system used for quantification of SAA.

In sheep infected with bacteria causing caseous lymphadenitis (CLA), serum Hp and SAA levels peaked by day 7 post-infection, which is considered to be the point at which the acute disease becomes a chronic infection [28]. Hp and SAA have also been previously reported to be measurable by day two post-infection in acute inflammatory diseases such as mastitis and metritis [16]. In contrast, the proportion of animals with a *P. ovis*-induced elevation in APPs did not occur until weeks 4-5 post-infestation in the case of Hp (Table 2), or marginally earlier at week 4 for SAA (Table 2). A possible explanation for this is that disease initiation in sheep scab has a “lag phase” at the start of infestation lasting for several weeks as the mites become established, after which the mite numbers increase exponentially [7]. Lesion size development follows this pattern in mite numbers, as the increasing numbers of mites move out from the margins of the lesion onto healthy skin, as shown by the lesion size data recorded in the TCT trial (Figure 1).

In order for a biomarker to be useful in the diagnosis of current disease status in sheep exposed to *P. ovis*, it is crucial that its levels in the serum decline rapidly after treatment or disease resolution. This study has demonstrated that Hp and SAA return to pre-infestation levels rapidly following termination of *P. ovis* infestation, with half-lives of 2.3 days for Hp and 0.84 days for SAA, illustrating the potential of including either or both of these APPs in an improved diagnostic test for sheep scab where knowledge of current disease status is important. For example, in the case of the Sheep Scab (Scotland) Order 2010, where confirmation of successful treatment is required before movement restrictions are lifted from the affected farm. The legislation allows lifting of movement restrictions to occur 16 days following successful treatment, when it is still difficult to assess by clinical examination. However, if Hp and /or SAA were used as BMs of current disease status, they would have returned to baseline levels before 16 days indicating successful treatment, as shown in Figure 5a and 5b.

SAA was a more accurate discriminatory indicator of current disease status in the PIT study than Hp, due to the higher sensitivity and specificity obtained for the optimised cut-off values which were estimated using the data from the primary infestation and post-treatment element of the PIT trial.

At the cut-off levels established for SAA and Hp by statistical analysis of the PIT experimental trial results, it was evident that, during secondary experimental infestation (i.e. re-infestation), mean Hp levels in the sera of infested sheep were below the Hp cut-off point at all time points. In addition, for SAA, mean sera levels were above the cut-off point for 1 week only during this secondary infestation period. This may reflect the altered immunological landscape during the pathogenesis of a re-infestation, where lesion size and mite numbers are reduced compared with primary lesions [5] and may represent a limitation for the use of BM assays although field outbreak studies are now required to establish suitable cut-off values in practice.

When Hp and SAA levels were investigated during other common diseases and conditions of sheep, they were predominantly found to be at levels equivalent to sheep scab negative values, remaining below 0.6 mg/mL for Hp and 22 μg/mL for SAA. Importantly, as gastro-intestinal nematodes (GIN) commonly affect sheep, Hp and SAA levels were not elevated during the GIN experimental infections tested here. Although Hp was elevated in liver fluke and Johnes disease and SAA was elevated in lice infestation in this analysis, it is evident from the standard errors in these results that there was large between animal variation and therefore these recorded measurements may be due to other underlying conditions. Analysis of additional sera samples from sheep infected with these diseases would therefore be required to confirm serum levels of Hp and SAA during these infections. In addition, as the BM assays react to other inflammatory conditions, they should be used in conjunction with the specific antibody assay which has been developed for sheep scab diagnosis and has been shown not to cross-react with other common sheep diseases and conditions, including biting lice [12]. Compared to Hp and SAA levels, the measurement of Pso o 2-specific IgG provided earlier, specific, diagnosis of *P. ovis* infestation as shown in Table 3, where the majority of animals showed an elevated antigen-specific IgG response to Pso o 2 in the primary infestation by week 2 post-infestation. This was a similar response to that previously reported using this assay [12]. While this assay therefore provides rapid, sensitive and specific diagnosis of *P. ovis* infestation, antibody persistence (Figure 5c and [13]) prevents accurate diagnosis of current disease status e.g. after treatment or on disease resolution. When the decreases in Hp and SAA levels post-treatment were compared to those of the Pso o 2 specific IgG levels, it was evident that Hp and SAA gave a rapid indication of current disease status post-treatment, whereas high Pso o 2-specific IgG levels were still measurable 20 weeks post-treatment. The differences in half life also illustrated the differing responses to treatment i.e. 56 days for the Pso o 2-specific IgG response, compared to 2.3 days for Hp and 0.84 days for SAA. However, as serum Hp and SAA levels increase in many inflammatory diseases in ruminants [16] these APPs could not be used in isolation for the diagnosis of sheep scab. The relative merits of the tests measuring the Pso o 2-specific IgG response and APP levels indicate that a combined diagnostic test incorporating both elements would result in a highly specific test which would indicate both pathogen specific early infestation (Pso o 2-specific IgG levels) and current disease status after treatment (SAA and/or Hp levels). This would provide the sheep industry with a powerful diagnostic tool for sheep scab control or eradication schemes.

## Competing interests

The authors declare they have no competing interests. PDE was a founding Director of ReactivLab Ltd and remains a shareholder and consultant.

## Authors’ contributions

Experimental design and planning: BW, AJN, STGB; Biomarker analyses: BW, EM, PDE; Statistical analyses BW, STGB, GI; Drafting of the manuscript: BW, AJN, STGB, PDE, GI. All authors read and approved the manuscript.
